# Semi-Annual Meeting of the Illinois State Veterinary Medical Association

**Published:** 1896-08

**Authors:** Albert Babb

**Affiliations:** Secretary


### SEMI-ANNUAL MEETING OF THE ILLINOIS STATE VETERINARY MEDICAL ASSOCIATION.

Union Hotel, Galesburg, Ill., May 19, 1896. In the absence of both President and Vice-President (Dr. M. R. Trumbower, President, and Dr. J. L. Tyler, Vice-President), Prof. A. H. Baker, of Chicago, was elected President *pro tem.*, and responded by an appropriate address.

The following members responded to roll-call: Drs. Albert Babb, A. H. Baker, F. L. Brown, E. S. Fry, T. J. Gunning, W. C. Hanawalt, J. McClintock, W. G. Neilson, R. P. Steddom, and John Scott.

After the Secretary made his report, Prof. A. H. Baker read his production, "Puncturing for Gastric Flatulence." The discussion of this valuable paper closed, on motion. The article of Dr. T. B. Newby, of Pana, entitled "An Anomalous Disease of the Horse," was read by the Secretary, the writer being absent. After the discussion of this paper the Association adjourned for dinner.

The Society reconvened at 2 P.M., and listened to the essay of Dr. John Scott on "Diseases and Injuries of the Foot." A lengthy discussion of this wide subject was finally closed, on motion.

A general and very interesting discussion then took place on "Barium Chloride as a Therapeutic Agent;" "Tuberculosis;" "Parturient Apoplexy;" Heat-exhaustion and Sunstroke in Horses;" "How to Fire for the Cure of Spavin;" and other meritorious topics.

On motion of Dr. Steddom, which was seconded by Dr. Gunning, Charles H. Herrick and Robert Gysel, both of the Chicago Veterinary College, class of '93, and R. C. Mylne, of the Montreal Veterinary College, class of '89, were elected to membership by acclamation.

The subject of State legislation was next taken up, and it was unanimously decided that the matter should be pushed to the front during the session of the coming legislature.

Adjourned to meet in Chicago in November.

ALBERT BABB, A.B., M.D.C.,
Secretary.

